# Uncommon Metastasis of Laryngeal Cancer to Small Bowel Causing Intestinal Obstruction Treated by Laparoscopic Approach

**DOI:** 10.1155/2014/260631

**Published:** 2014-09-01

**Authors:** Omar Bekdache, Lateefa Al Nuaimi, Haytham El Salhat, Vasudev Sharma, Ghodratollah Nowrasteh, Sadir J. Al Rawi

**Affiliations:** ^1^Division of Surgical Oncology, Department of Surgery, Tawam Hospital Affiliated to Johns Hopkins Medicine, Al Ain, UAE; ^2^Department of Pathology, Tawam Hospital Affiliated to Johns Hopkins Medicine, Al Ain, UAE

## Abstract

Metastatic laryngeal cancer to the small bowel is extremely rare. Management of small bowel obstruction used to constitute a relative contraindication for the use of laparoscopic modality. We are reporting a case of an elderly man known to have laryngeal cancer who presented with small bowel obstruction due to metastatic deposit to the small bowel. The condition was successfully treated by laparoscopic assisted approach. A review of the natural history of advanced laryngeal cancer, common and uncommon sites of metastasis, and the rare presentation as small bowel obstruction is illustrated in this review.

## 1. Introduction

Complete small bowel obstruction secondary to metastatic laryngeal caner is rarely reported in the literature. Advanced laryngeal squamous cell carcinoma usually spread via lymphatic channels to the neck and chest. Most common sites of metastasis are reported to be in the lungs. In the rare conditions where the hematogenous spread of the disease leads to small bowel deposit, it may manifest itself as small bowel obstruction due to constricting involvement of the bowel wall by the neoplastic process. Other rare presentations of small bowel involvement include hemorrhage and perforation.

Treatment of the condition is most of the time carried out on emergency basis with resection of the involved bowel segment using conventional techniques. In order to be able to use minimally invasive modality, a working space is usually required. This space is often obscured by the dilated bowel loops secondary to the obstruction, which make the performance of such procedures very challenging. The following case illustrates the successful treatment of the rare metastatic laryngeal cancer to the small bowel using laparoscopic assisted techniques.

## 2. Case Report

An 82-year-old Emirati male was diagnosed to have laryngeal cancer in 2007. He underwent surgical treatment with total laryngectomy and adjuvant chemoradiotherapy for a Stage III disease. Follow-up by Ear, Nose and Throat colleagues showed no evidence of recurrence over 6 years. He is well surrounded by very caring family members and was able to manage his daily activities with little help.

One month prior to his presentation to the hospital, the patient reported intermittent constipation associated with vague mild abdominal pain. Three weeks later, the patient presented to the emergency department complaining of obstipation for the last three days, nausea, and two episodes of biliary vomiting, mild abdominal distention, and exacerbation of the diffuse colicky abdominal pain. Pertinent physical findings revealed a cooperative patient with an ECOG performance status of 2, dehydrated with dry mucous membranes, a soft abdomen with moderate distention mainly in the upper part, tympanic to percussion, with slight exaggeration of the small bowel sounds on auscultation. Rectal examination showed an empty rectum. His vital signs were normal. He was not known to have previous hypertension, diabetes, or other cardiac illnesses, so aggressive parenteral hydration concurrently with the request of basic laboratory and radiological tests was performed. Laboratory tests showed no gross abnormalities beside a slight elevation of his hematocrit level that was attributed to the dehydration status.

**Figure 1:**
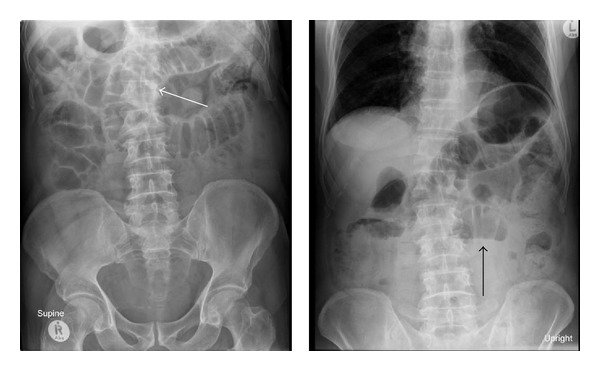
Supine and upright plain abdominal films showing distended small bowel loops (white arrow) and scant air-fluid levels (black arrow).

Abdominal films showed marked dilatation of the small bowel loops on the supine film and scant air fluid levels on the upright position (Figure 1).

**Figure 2:**
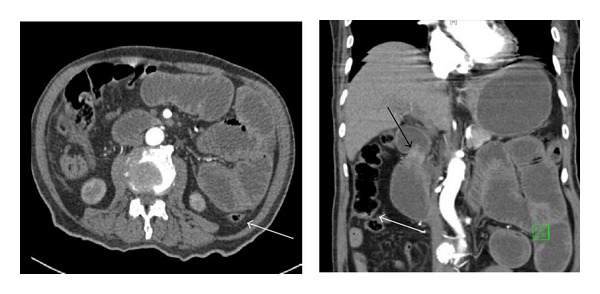
CT scan with oral and intravenous contrast showing dilated small bowel loops with edematous walls. Collapsed distal small bowel and colon is also noted (white arrow). A possible transition area in the small bowel loops was appreciated (black arrow).

With previous virgin abdomen and with no obvious hernia, CT scan with oral and intravenous contrast was requested. It revealed the presence of dilated small bowel loops with edematous walls, mainly occupying the upper and mid-abdomen. Collapsed distal small bowel loops and normal colonic diameter were also noted. A possible transition area in the small bowel loops was appreciated without obvious intra- or extraluminal pathology (Figure 2).

**Figure 3:**
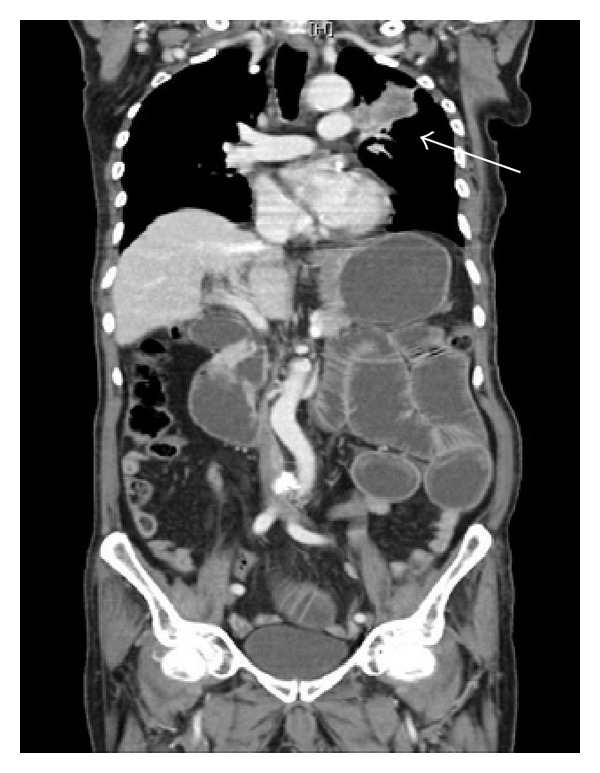
Mass lesion localized in the upper lobe of the left lung and encasing the origin of the left pulmonary artery (white arrow).

Computed tomogram of the chest showed as well the presence of large mass occupying lesion localized in the upper lobe of the left lung and encasing the origin of the left pulmonary artery (Figure 3).

A nasogastric tube and a urinary catheter were inserted and accurate intake and output measurement were instituted. Resuscitation was carried out for 24 hours. A decision to take him to the operating room was made, and due to the moderate distention of the abdomen, an attempt to proceed with minimal invasive approach was contemplated.

**Figure 4:**
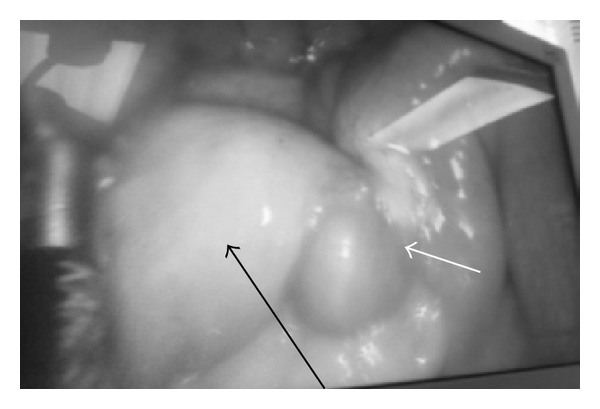
Laparoscopic view of the constricting whitish nodule obstructing the intestinal lumen (white arrow) with proximal dilatation of the bowel (black arrow).

Extra caution was used while insufflating the abdominal cavity using a classical supraumbilical approach to prevent an inadvertent injury to the dilated small bowels. Laparoscopic exploration was made through a 5 mm 30 degree scope introduced through a 5 mm trocar. It revealed the presence of significant dilatation of small bowel loops. No disseminated disease or liver involvement was notified. Assisted by another two trocars inserted in the left lower quadrant and mid-way between the umbilicus and symphysis pubis, and helped by a reversed Trendelenburg position, the bowel was traced from the duodenojejunal junction downward. About 75 cm from the ligament of Treitz, a constricting whitish nodule was seen obstructing. Collapsed distal loops and a normal caliber colonic frame were noted (Figure 4).

**Figure 5:**
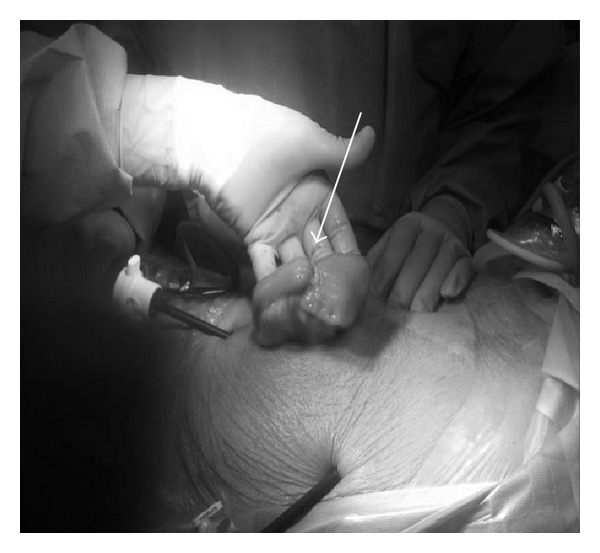
Diseased bowel segment exteriorized through a 4 cm skin incision with obvious discrepancy between the proximal and distal bowel diameters (white arrow).

The collapsed bowel distal to the nodular area was gently grasped using a nontraumatic grasper. The umbilical port site was enlarged to about a 4 cm incision, and the diseased bowel segment was exteriorized through the skin (Figure 5). A 5 cm healthy margin from each side and a triangular mesenteric wedge were excised. Functional end-end anastomosis was fashioned using stapling technique.

The minilaparotomy was closed tightly after reintroduction of the bowel in the peritoneal cavity and the abdomen was reinsufflated again to assure normal configuration of the abdominal contents. Total operative time was around 40 minutes. The patient made an uneventful postoperative recovery except for superficial umbilical site wound infection and was able to regain normal bowel habits as soon as the second postoperative day.

**Figure 6:**
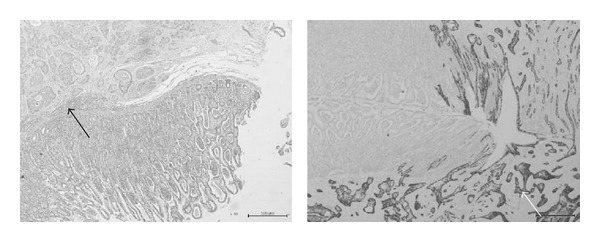
Histopathological slides showing normal small bowel mucosa with tumor invasion from the serosa reaching the submucosa on hematoxylin and eosin stains (black arrow). Typical keratinization of squamous cell carcinoma on the immunohistochemistry stain, tumor cells are positive for CK5/6 and negative for Napsin A (white arrow) confirming squamous cell cancer of laryngeal origin.

Histopathology and immunohistochemistry proved to be squamous cell cancer. Napsin A test was negative and favors the metastatic deposit to be of laryngeal origin (Figure 6).

## 3. Results

Laryngeal cancer contributes to about 2% of malignant fatalities. There was a slight increase in its incidence due to tobacco-alcoholic abuse in both genders [1].

Squamous cell pattern constitutes the vast majority of laryngeal carcinomas. The main modality of disease spread is made via lymphatic pathways especially in the supraglottic location of the disease that is richly invested by lymphatic channels which crosses the midline, in contradistinction to the glottic and infraglottic counterparts. Hence, supraglottic cervical lymph node metastasis can be seen in up to 40% of the cases. Early glottic cancer carries a good prognosis, with 3-year tumor-free survival reaching 90%. On the other hand, advanced supraglottic 3-year tumor-free survivals is estimated to be around 40% [2].

Distant metastasis to other organs is made via hematogenous spread. Several hypotheses were raised to explain very atypical sites of metastasis that are away of the norm of a specific tumor spread, among these a possible alternate pathway of spread induced by surgery or radiation therapy, but none of these could have been proven. The most common organ involved by distant metastasis from laryngeal primary is the lung. In addition to sites like liver and bone, unusual sites of sporadic distant metastasis were reported in the literature to involve the skin [3], forearm [4], heart [5], kidney [6], and genitalia [7].

Small bowel involvement by neoplastic secondaries occurs rarely, but still it outnumbers primary small bowel tumors. It can manifest itself through gastrointestinal bleeding [8], perforation, intussusception, or obstruction [9, 10]. In case of intestinal obstruction, most of the patients will be operated on on emergency basis, and the origin of metastasis will not be discovered until definitive pathological examination is performed. A similar case of complete small bowel obstruction caused by metastasis from primary head and neck carcinoma was recently reported from the Department of Clinical Oncology, Prince of Wales Hospital in the Chinese University of Hong Kong as being the first case to be published in the literature; the difference is that the primary neoplasm in the reportable case was located in the nasopharynx [11].

A recent review of all published cases of SCC from head and neck cancer sites by Dwivedi et al. revealed only 12 such cases, of which only nine cases were derived from a laryngeal primary neoplasm. In their review they described three cases in which a laryngeal SCC metastasis caused intestinal obstruction, two cases of bleeding or melena, one case of biliary obstruction, and three cases of small bowel perforation [12]. Differentiating the origin of metastatic squamous cell cancer can be difficult. In a review of more than 190 cases, immunohistochemistry tests can be of help, as the negativity of Napsin A test can be in favor of the non-pulmonary origin of the metastatic squamous cell cancer, and in our case, the laryngeal origin [13].

Of interest, intestinal obstruction used to be a relative contraindication for laparoscopic surgery mainly due to the loss of the virtual space that is usually created by the institution of pneumoperitoneum. In case of obstruction, this extensible space will be occupied by the distended obstructed bowel loops. Another hazard that made minimal invasive surgery shies away from treating intestinal obstruction is the higher probability of iatrogenic small bowel injuries, due to adhesions from previous operations, which constitute the most common cause of small bowel obstruction. Recent research done about the feasibility of laparoscopy in the management of small bowel obstruction concluded at the safety of the modality if it is carried out by experienced hands [14].

## 4. Conclusion

The specificity of this case comes from two distinctive facts. The first is being the rarity of metastatic laryngeal primary to manifest itself as isolated complete small bowel obstruction. The second fact was the ability to manage the condition using minimal invasive techniques, the thing that reflected positively on the recovery of the patient with subsequent earlier return to his preoperative condition.

Small bowel obstruction, especially with a negative previous abdominal surgical history and in a patient with known malignant disease elsewhere in the body, should raise the suspicion that both conditions could be related.
